# Mechanical Complications After Acute Myocardial Infarction: Contemporary Management, Treatment Selection, and Outcomes in a 10-Year Regional Referral Network

**DOI:** 10.3390/jcm15207975

**Published:** 2026-10-15

**Authors:** Alberto Alén, Andrés Álvarez-Gato, Manuel Sánchez, Francisco González-Urbistondo, María Salgado, Rut Álvarez-Velasco, Miguel Ángel Rodríguez-Yago, María Isabel Soto, Jacobo Silva, Pablo Avanzas, Rebeca Lorca

**Affiliations:** 1 Cardiology, Hospital Universitario Central de Asturias (HUCA), 33011 Oviedo, Spain; 2 Instituto de Investigación del Principado de Asturias (ISPA), 33011 Oviedo, Spain; 3 Cardiology, Hospital de Sierrallana, 39300 Torrelavega, Spain; 4 Cardiology, Hospital Universitario Marqués de Valdecilla, 39008 Santander, Spain; 5 Cardiac Surgery Critic Care Unit, Hospital Universitario Central de Asturias (HUCA), 33006 Oviedo, Spain; 6 Cardiac Surgery, Hospital Universitario Central de Asturias (HUCA), 33006 Oviedo, Spain; 7 Faculty of Medicine, University of Oviedo, 33006 Oviedo, Spain

**Keywords:** acute myocardial infarction, mechanical complications, ventricular septal rupture, free-wall rupture, papillary muscle rupture, mechanical circulatory support, cardiac surgery

## Abstract

**Background:** Mechanical complications (MCs) after acute myocardial infarction (AMI) are now uncommon but remain highly lethal, and contemporary data encompassing the full spectrum of rupture phenotypes are limited. **Methods:** We retrospectively identified all consecutive adults with post-AMI MCs managed at a tertiary regional referral center from 2015 to 2025. MCs included ventricular septal rupture (VSR), left ventricular free-wall rupture (LVFWR), contained rupture/pseudoaneurysm, and papillary muscle rupture (PMR). The primary endpoint was 30-day all-cause mortality. Exploratory analyses assessed treatment patterns by age, sex, geographical distance, and area-level income. **Results:** Among 59 patients (mean age 71.2 ± 10.7 years; 50.8% women), 17 (28.8%) had isolated VSR, 21 (35.6%) LVFWR, 10 (16.9%) combined VSR and contained LVFWR/pseudoaneurysm, and 11 (18.6%) PMR. Temporary mechanical circulatory support (MCS) and surgery were each used in 35 patients (59.3%). Thirty-day mortality was 59.3%. Among patients undergoing definitive invasive treatment, mortality was 45.7%. Older age was associated with higher 30-day mortality (OR per year 1.09, 95% CI 1.03–1.16), whereas surgery was associated with lower observed mortality (OR 0.22, 95% CI 0.07–0.73), acknowledging major treatment selection and survivor biases. Patients aged ≥75 years received MCS less frequently than younger patients (29.2% vs. 80.0%), and women underwent surgery less frequently than men (46.7% vs. 72.4%). Distance and area-level income were not associated with 30-day mortality. **Conclusions:** In a contemporary regional referral network, post-AMI MCs remained associated with extreme early mortality. Age and treatment selection were major determinants of observed outcomes, while marked age- and sex-related differences in treatment intensity warrant confirmation in larger multicenter cohorts.

## 1. Introduction

Mechanical complications (MCs) are among the most catastrophic consequences of acute myocardial infarction (AMI). The classic spectrum comprises ventricular septal rupture (VSR), left ventricular free-wall rupture (LVFWR), contained rupture or ventricular pseudoaneurysm, and acute severe mitral regurgitation caused by papillary muscle rupture (PMR) [1,2]. Their incidence has fallen substantially with early reperfusion and primary percutaneous coronary intervention (PCI), but their lethality has not declined to the same extent [3].

In a contemporary US analysis of more than nine million AMI admissions, MCs occurred in 0.27% of ST-segment elevation myocardial infarctions with in-hospital mortality of 42.4% [3]. Current ESC guidelines therefore emphasize immediate echocardiographic assessment when a mechanical complication is suspected and prompt referral for specialized management [4].

Definitive treatment is particularly challenging because the clinical course combines profound hemodynamic instability with friable infarcted myocardium. Surgery remains the principal definitive treatment for most MCs, while percutaneous repair has an increasing role in selected VSRs and other anatomically suitable lesions [1,5]. For post-infarction VSR, observational studies consistently show high mortality with early repair, whereas patients who can be stabilized and undergo delayed intervention appear to have better outcomes [6,7]. These comparisons are, however, strongly affected by survivor selection and immortal-time bias.

Temporary mechanical circulatory support (MCS) may provide a bridge to definitive therapy by stabilizing systemic perfusion and allowing time for multidisciplinary assessment and tissue maturation [1,7]. Evidence remains predominantly observational, and device selection must account for the specific physiology of each mechanical defect.

Contemporary evidence is dominated by administrative datasets or lesion-specific surgical series, leaving limited real-world information on the complete spectrum of post-AMI MCs within a single regional system of care. Important uncertainties also remain regarding treatment selection in older adults and women and whether regional organization can attenuate geographical or socioeconomic barriers. We therefore evaluated a 10-year, all-comers cohort encompassing the full anatomical spectrum of post-AMI MCs managed within a tertiary regional referral network. Our objectives were to characterize contemporary phenotypes and treatment pathways, identify factors associated with 30-day mortality, describe long-term mortality, and explore differences in treatment according to age, sex, geographical distance, and socioeconomic status.

## 2. Materials and Methods

### 2.1. Study Design and Population

We performed a retrospective, observational, all-comers cohort study including consecutive adult patients diagnosed with an MC after AMI and managed at a tertiary cardiovascular referral center between 2015 and 2025. The center is the regional hub for cardiac surgery, advanced cardiac critical care, interventional cardiology, and temporary MCS within a *hub-and-spoke* referral network. Eligible MCs were LVFWR (free or contained), isolated VSR, combined VSR with contained LVFWR/pseudoaneurysm, and acute severe mitral regurgitation secondary to PMR. Cases were identified from institutional clinical records by discharge codes and individually reviewed to confirm the anatomical diagnosis.

### 2.2. Definitions and Data Collection

Demographic characteristics, cardiovascular risk factors, comorbidities, infarct location, coronary angiography and revascularization, clinical presentation, management, and outcomes were retrospectively collected from electronic medical records. LVFWR was categorized as free rupture or contained rupture/pseudoaneurysm; PMR was classified by papillary muscle involvement when available. Temporary MCS was defined as use of any short-term circulatory support device during the index episode. Definitive treatment included surgical repair/replacement or other lesion-directed intervention. Early VSR repair was defined as surgery performed within 5 days of MC diagnosis. Geographical distance was calculated from the patient’s area of residence to the tertiary referral center, and socioeconomic status was assessed using area-level median income.

### 2.3. Outcomes

The primary endpoint was 30-day all-cause mortality, defined as death from any cause within 30 days after onset/diagnosis of the mechanical complication. Long-term all-cause mortality at the last available follow-up was a secondary endpoint. Exploratory analyses evaluated use of temporary MCS and definitive surgery according to age and sex. Patients aged ≥75 years were prespecified as an older subgroup.

### 2.4. Statistical Analysis

Continuous variables are reported as mean ± standard deviation or median [interquartile range], according to distribution, and categorical variables as number (percentage). Between-group comparisons used Student’s *t* test or the Mann–Whitney U test for continuous variables and the chi-square or Fisher exact test for categorical variables, as appropriate. Univariable logistic regression was used to estimate associations with 30-day all-cause mortality and is reported as odds ratios (ORs) with 95% confidence intervals (CIs). Given the limited sample size and number of events, no multivariable models were performed to avoid overfitting. Accordingly, regression analyses were considered exploratory, and no independent or causal treatment effects were inferred. Long-term survival was analyzed using Kaplan–Meier methods. All tests were two-sided, and *p* < 0.05 was considered statistically significant. Analyses were performed using IBM SPSS Statistics version 30.0.0.0.

### 2.5. Ethics

The study was conducted in accordance with the Declaration of Helsinki and was approved by the competent institutional research ethics committee (CEImPA 2026.199). The waiver of informed consent was included, as stipulated in the study protocol. Generative AI-assisted tools were used exclusively for graphical refinement of the Central Illustration. All numerical and clinical content was verified by the authors.

## 3. Results

### 3.1. Cohort and Mechanical Complication Phenotypes

**Figure 1:**
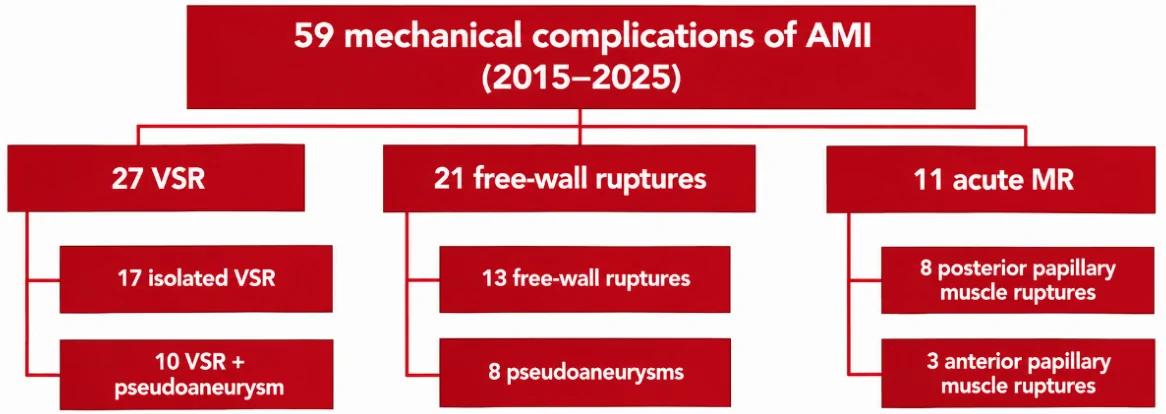
Distribution of mechanical complications after acute myocardial infarction. Flow diagram showing the distribution of mechanical complications according to phenotype and anatomical subtype among the 59 patients included in the study. AMI, acute myocardial infarction; VSR, ventricular septal rupture; MR, mitral regurgitation.

A total of 59 patients with post-AMI MCs were identified (Figure 1). Mean age was 71.2 ± 10.7 years; 29 (49.2%) were men and 30 (50.8%) were women (Table 1). Hypertension was present in 26 (44.1%), dyslipidemia in 21 (35.6%), and diabetes mellitus in 11 (18.6%). The index infarction was inferior in 30 (50.8%), anterior in 23 (39.0%), posterior in four (6.8%), and non-ST-segment elevation AMI in two (3.4%). Isolated VSR occurred in 17 patients (28.8%), LVFWR in 21 (35.6%), combined VSR and contained LVFWR/pseudoaneurysm in 10 (16.9%), and PMR in 11 (18.6%). Thus, VSR was present in 27 patients (45.8%) when isolated and combined phenotypes were considered together. Among PMR cases, eight involved the posteromedial and three the anterolateral papillary muscle.

### 3.2. Mechanical Circulatory Support and Definitive Treatment

Temporary MCS was used in 35 patients (59.3%). IABP was the predominant support strategy and was used in 33 of 35 MCS-treated patients (94.3%): as isolated support in 23 patients, combined with VA-ECMO in nine, and combined with CentriMag in one. VA-ECMO alone was used in one patient and Impella alone in one patient. Overall, 12 patients (20.3%) received advanced MCS beyond isolated IABP. Patients receiving advanced MCS were younger than those managed without MCS (63.7 ± 8.7 vs. 76.2 ± 10.8 years; *p* = 0.002). Definitive surgery was performed in 21/23 (91.3%) patients treated with IABP alone, 7/12 (58.3%) receiving advanced MCS, and 7/24 (29.2%) receiving no MCS (*p* < 0.001). These unadjusted differences should be interpreted descriptively, given the marked differences in age, clinical presentation, mechanical complication phenotype, and likelihood of reaching definitive surgical treatment across support strategies.

**Figure 2:**
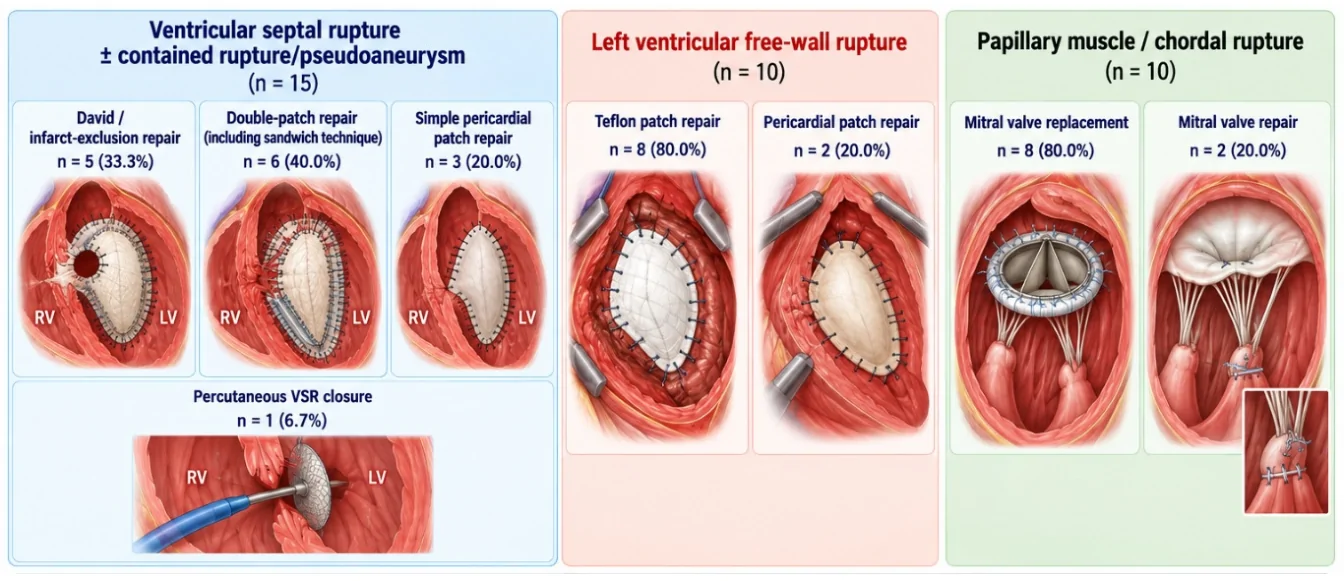
Surgical techniques used for the repair of mechanical complications after acute myocardial infarction. Schematic representation of the main surgical strategies employed in the cohort, including David/infarct-exclusion repair, double-patch repair and simple pericardial patch repair for ventricular septal rupture; patch repair for left ventricular free-wall rupture; and mitral valve replacement or repair for papillary muscle or chordal rupture. AMI, acute myocardial infarction; LV, left ventricular; VSR, ventricular septal rupture.

Thirty-five patients (59.3%) underwent definitive invasive treatment (Figure 2). Among the 15 patients with VSR or combined VSR/contained rupture undergoing definitive invasive treatment, five underwent David/infarct-exclusion repair, six double-patch repair (“*sandwich*” technique), three simple pericardial patch repair, and one percutaneous VSR closure. Among the 15 patients who underwent surgical treatment, two underwent early surgery, whereas the remaining 13 were operated on after a delay of 5 days or more. All 10 treated patients with LV free-wall rupture underwent patch repair, using Teflon in eight and pericardium in two. Among the 10 patients treated for papillary muscle or chordal rupture, mitral valve replacement was performed in eight and mitral valve repair in two. Concomitant coronary artery bypass grafting was performed in five patients. Overall, 30-day mortality among patients undergoing definitive invasive treatment was 45.7% (16/35) and 40.0% (6/15) among patients undergoing surgical repair of VSR. Three patients (5.1%) were listed for urgent heart transplantation.

### 3.3. Thirty-Day Mortality

Thirty-five patients died within 30 days, yielding a 30-day all-cause mortality of 59.3%. Patients who died were older than 30-day survivors (74.7 ± 9.5 vs. 66.0 ± 10.2 years; *p* = 0.002). Thirty-day mortality did not differ significantly by sex (63.3% in women vs. 55.2% in men; *p* = 0.524) or by mechanical complication phenotype (global *p* = 0.717). Mortality was 79.2% among patients who did not undergo definitive invasive treatment and 45.7% among those who did (unadjusted OR 0.22; *p* = 0.010). However, this association should be interpreted cautiously given the potential for confounding by indication and immortal-time bias and should not be considered evidence of a causal protective effect of surgery. Temporary MCS, considered as any support modality in the current database, was not significantly associated with 30-day mortality (51.4% with MCS vs. 70.8% without MCS; *p* = 0.136).

In univariable logistic regression, each additional year of age was associated with higher odds of 30-day mortality (OR 1.09, 95% CI 1.03–1.16; *p* = 0.004). Surgery was associated with lower odds of 30-day mortality (OR 0.22, 95% CI 0.07–0.73; *p* = 0.013).

### 3.4. Age-Related Treatment Patterns

Twenty-four patients (40.7%) were aged ≥75 years. Thirty-day mortality was 70.8% in patients aged ≥75 years compared with 51.4% in younger patients (*p* = 0.136). Older patients received temporary MCS substantially less often (29.2% vs. 80.0%; *p* < 0.001) and underwent surgery numerically less often (45.8% vs. 68.6%; *p* = 0.081). Given the limited sample size, these subgroup analyses should be considered exploratory and hypothesis-generating rather than confirmatory.

### 3.5. Sex-Related Treatment Patterns

Men and women did not differ significantly in age (68.9 ± 11.2 vs. 73.4 ± 9.7 years; *p* = 0.102), and baseline characteristics were otherwise broadly comparable between groups. Men were more frequently treated surgically than women (21/29 [72.4%] vs. 14/30 [46.7%]; *p* = 0.044), whereas the use of temporary mechanical circulatory support was similar between sexes (65.5% vs. 53.3%; *p* = 0.341). Despite the higher rate of surgical treatment among men, 30-day mortality was comparable between women and men (55.2% vs. 63.3%; *p* = 0.524). Long-term survival also did not differ (Supplementary Material Figure S1) significantly by sex (log-rank *p* = 0.344). Given the limited sample size, these subgroup analyses should be considered exploratory and hypothesis-generating rather than confirmatory.

### 3.6. Long-Term Outcomes

**Figure 3:**
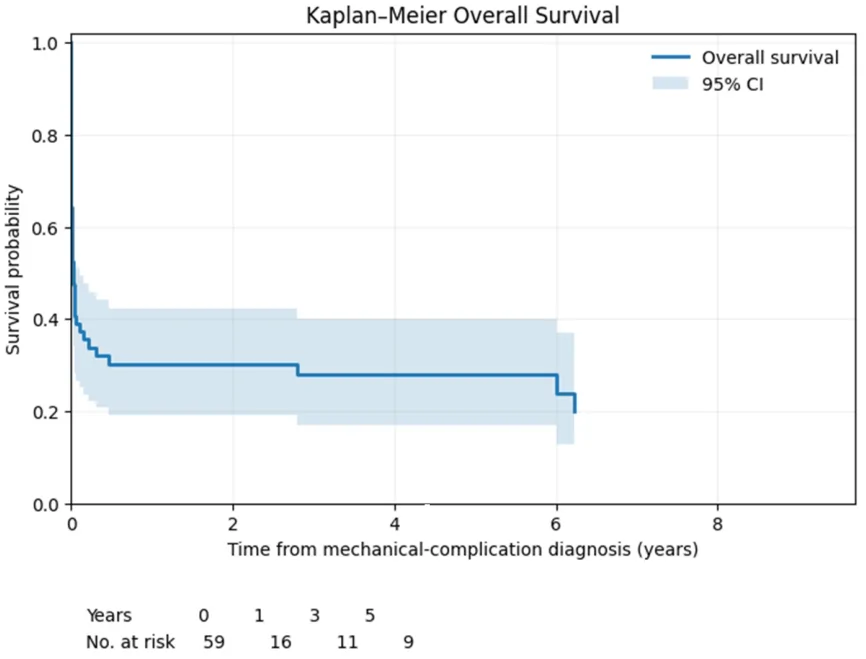
Long-term survival after mechanical complications of acute myocardial infarction. Kaplan–Meier estimate of overall survival from the diagnosis of the mechanical complication. Shaded areas represent 95% confidence intervals. Numbers at risk are shown below the graph. AMI, acute myocardial infarction; CI, confidence interval.

Follow-up was closed in May 2026, with complete vital status ascertainment for all 59 patients. During follow-up, 44 patients (74.6%) died. Overall Kaplan–Meier survival was 30.3% (95% CI 19.1–42.2%) at 1 year, 28.0% (95% CI 17.0–40.0%) at 3 years, and 28.0% (95% CI 17.0–40.0%) at 5 years (Figure 3). No significant differences in long-term survival were observed between men and women (log-rank *p* = 0.401).

### 3.7. Geographical and Socioeconomic Factors

Mean distance from residence to the tertiary referral center was 28.7 ± 23.1 km, and median area-level income was EUR 15,591.5. Neither distance nor income differed significantly between 30-day survivors and non-survivors (Mann–Whitney *p* = 0.666 and *p* = 0.324, respectively). Previous regional analyses also found no significant differences in diagnostic delay, presentation, or surgery according to place of residence.

## 4. Discussion

This 10-year all-comers regional cohort provides a contemporary view of the full spectrum of post-AMI MCs within an organized referral network. Four findings merit emphasis. First, early mortality remained extreme: nearly six in 10 patients died within 30 days despite access to surgery, advanced critical care, and temporary MCS. Second, advanced age was the clearest patient-level marker of early mortality. Third, treatment intensity differed substantially across clinically relevant groups: patients aged ≥75 years received MCS far less frequently, while women underwent surgery less often than men. Fourth, neither geographical distance nor area-level income was associated with early mortality after referral. These findings reinforce that contemporary MCs are rare but exceptionally lethal and suggest that outcomes reflect not only anatomy and shock severity but also candidacy, timing, bridging strategies, and the organization of regional care.

The incidence of post-AMI MCs has fallen to well below 1% in contemporary reperfusion systems [1,2,3,4], yet mortality remains strikingly high. In a large US analysis including almost 9 million AMI hospitalizations, Elbadawi et al. reported MCs in 0.27% of STEMI and 0.06% of NSTEMI admissions, with in-hospital mortality rates of 42.4% and 18.0%, respectively [3]. Importantly, mortality did not meaningfully improve over the 13-year study period despite changes in reperfusion and increasing availability of advanced MCS. These observations have subsequently been reinforced by contemporary reviews and scientific statements emphasizing that successful primary prevention of myocardial rupture through early reperfusion has not been matched by a comparable improvement in prognosis once a structural complication has occurred [8,9,10]. Delayed diagnosis may also contribute to the persistently high mortality associated with mechanical complications, underscoring the importance of prompt bedside echocardiography, including transthoracic and, when required, transesophageal imaging, in acute cardiac care to facilitate early diagnosis and timely intervention [11]. Our 59.3% 30-day mortality is higher than that reported in the overall STEMI population with MCs by Elbadawi et al., plausibly reflecting referral enrichment for the most severe cases, inclusion of free-wall rupture, and the presence of combined structural phenotypes. The cohort therefore illustrates the central paradox of modern MCs: they have become increasingly rare but remain disproportionately lethal.

LVFWR accounted for 35.6% of cases, isolated VSR for 28.8%, combined VSR and contained free-wall rupture/pseudoaneurysm for 16.9%, and PMR for 18.6%. The 10 patients with combined septal and free-wall involvement are clinically relevant. In the large CAUTION surgical VSR cohort, concomitant post-infarction mechanical complications were documented in approximately 5% of patients, confirming that rupture does not invariably respect a single anatomical compartment [12]. The necrotic process after a transmural infarction may involve contiguous septal and free-wall myocardium, and the resulting phenotype can evolve dynamically from intramyocardial dissection or contained rupture to overt septal or free-wall communication. Accordingly, contemporary JACC reviews emphasize systematic assessment for associated structural lesions whenever one MC is identified [8,9]. Our relatively high proportion of combined lesions may also reflect detailed imaging and surgical characterization at a tertiary referral center and argues against treating VSR, LVFWR and pseudoaneurysm as entirely isolated entities.

The particularly high representation of LVFWR also deserves consideration. Free-wall rupture is among the most catastrophic manifestations of myocardial necrosis and may cause sudden death before hospital evaluation, creating substantial survivor and referral bias in all clinical series. Contemporary estimates place overt LVFWR at only approximately 0.01–0.02% of AMI, although its true incidence is difficult to establish because rapidly fatal cases may never reach definitive imaging or surgery [8]. Clinical presentation encompasses a spectrum from abrupt blowout rupture with massive hemopericardium and electromechanical dissociation to slower oozing rupture or contained rupture with pseudoaneurysm formation. This distinction has therapeutic and prognostic implications. In the multicenter CAUTION analysis of 140 surgically treated LVFWR patients, operative mortality remained 36.4%, and cardiac arrest, lower preoperative LVEF, female sex, and need for extracorporeal support were independently associated with mortality [13]. The persistence of substantial mortality even among patients who reach surgery underscores that the observed burden in hospital cohorts represents only the visible component of an even more lethal complication.

Surgery remains the principal definitive therapy for most post-AMI MCs. Current ACS guidelines recommend management in centers with cardiac surgical expertise and consider short-term MCS reasonable for hemodynamic stabilization as a bridge to definitive treatment [10]. In our cohort, operated patients had lower observed 30-day mortality. This association is clinically plausible but cannot be interpreted causally: surgical candidacy depends on anatomy, shock severity, comorbidity, frailty, neurological status, technical feasibility, and survival long enough to reach the operating room. Accordingly, comparisons between surgical and conservative management are vulnerable to confounding by indication and immortal-time bias.

The question of timing is particularly important in VSR. Immediate repair may be unavoidable in patients with refractory shock, but operating on acutely necrotic and friable myocardium increases the technical difficulty of anchoring a patch and the risk of residual or recurrent shunting. Conversely, postponing repair may permit infarct maturation and fibrosis but exposes the patient to continued left-to-right shunting, pulmonary overcirculation, right ventricular failure, progressive multiorgan dysfunction, and sudden hemodynamic collapse. In a Spanish multicenter cohort of 141 patients, conservative management was associated with 91.5% mortality compared with 52.3% among surgically treated patients, and the lowest surgical mortality was observed when repair could be deferred until day 4 or later [7]. Similarly, the international CAUTION study of 475 surgically treated VSR patients reported 40.4% early mortality, with older age, cardiac arrest and markers of severe perioperative circulatory failure associated with adverse outcomes; patients undergoing later repair had better observed survival [12]. Historical data from the Society of Thoracic Surgeons database similarly showed markedly higher operative mortality when repair was undertaken within 7 days of infarction [6]. These remarkably consistent observational associations support stabilization and delayed repair when physiologically feasible, but they should not be interpreted as evidence that delaying surgery itself improves survival. Patients capable of surviving several days without repair constitute an intrinsically selected lower-risk population, whereas the sickest patients require immediate intervention or die before delayed surgery becomes possible.

### 4.1. Mechanical Circulatory Support

Temporary MCS was used in 59.3% of patients, underscoring the severity of hemodynamic compromise and the importance of bridging strategies in contemporary practice [13]. The physiological objective of MCS in MCs differs from routine cardiogenic shock support because each device modifies ventricular loading conditions, trans-septal pressure gradients, pulmonary blood flow, and myocardial wall stress. This is particularly relevant in VSR. Intra-aortic balloon counterpulsation can reduce afterload and shunt magnitude but provides limited augmentation of systemic flow. Direct LV unloading with microaxial flow pumps may reduce LV pressure, pulmonary capillary wedge pressure, and left-to-right shunting, whereas peripheral VA-ECMO restores systemic perfusion but may increase LV afterload and, without adequate unloading, potentially augment shunt flow and pulmonary congestion [7]. Device selection therefore cannot be separated from the underlying structural physiology.

Evidence supporting advanced MCS remains predominantly observational. Ronco et al. reviewed 111 studies comprising 2440 patients with post-infarction VSR; almost all received IABP, while advanced MCS was reported in a much smaller subset, predominantly VA-ECMO. Mean bridging duration was approximately 6 days, and overall in-hospital mortality remained approximately 50% [6,7]. The apparent favorable outcomes reported in selected ECMO-supported patients are hypothesis-generating rather than comparative evidence because device allocation is profoundly influenced by shock severity, institutional expertise and candidacy for definitive repair. Indeed, large administrative datasets have not demonstrated a population-level mortality reduction associated with MCS use [3]. MCS should therefore be viewed primarily as a strategy to create time—time to reverse hypoperfusion, assess neurological prognosis, define anatomy, transfer to an experienced center, and potentially allow myocardial tissue to mature before repair—rather than as definitive therapy itself.

### 4.2. Age and Treatment Selection

Patients aged ≥75 years constituted 40.7% of the cohort and experienced 70.8% 30-day mortality. Advanced age has repeatedly emerged as an adverse prognostic marker in post-AMI MCs. In the US analysis by Elbadawi et al., mortality among STEMI patients with MCs was 54.3% in those aged >75 years compared with 35.6% in younger patients [3]. Our results therefore extend a consistent observation from much larger datasets to a real-world regional population with a substantial representation of elderly patients.

Particularly striking, however, was the markedly lower use of MCS among patients aged ≥75 years. This finding should not automatically be interpreted as undertreatment. Chronological age integrates several dimensions that were incompletely captured in our database, including frailty, functional status, cognitive impairment, comorbidity burden, vascular access limitations, probability of meaningful recovery, operative candidacy, and patient preferences. Furthermore, MCS is most rational when it bridges toward a realistic therapeutic destination; advanced support may provide little benefit when definitive repair is not considered feasible. Nevertheless, the magnitude of the age-related difference suggests that treatment intensity in elderly patients with MCs warrants specific investigation. Future registries should capture frailty, baseline functional status, treatment limitation decisions and reasons for withholding surgery or MCS to distinguish appropriate selection from potentially modifiable age-related differences in care.

### 4.3. Papillary Muscle Rupture

PMR represents a distinct physiological phenotype because abrupt loss of mitral competence can generate torrential regurgitation, pulmonary edema and shock despite a relatively preserved or hyperdynamic LVEF. Contemporary data estimate PMR itself to occur in approximately 0.05% of AMI, most commonly involving the posteromedial papillary muscle because of its more limited coronary blood supply [14]. Surgery remains the standard definitive treatment, although transcatheter edge-to-edge repair is emerging as a rescue option in selected patients considered prohibitive surgical candidates [15]. The international CAUTION PMR cohort included 214 surgically treated patients and reported an in-hospital mortality of 24.8%, with mitral valve replacement performed in more than 80% [16]. A subsequent meta-analysis including 1851 surgically treated patients estimated operative mortality at approximately 21%, demonstrating that PMR remains highly lethal despite generally better surgical outcomes than those observed with VSR or LVFWR [5]. These phenotype-specific differences reinforce the importance of reporting the anatomical composition of MC cohorts rather than treating all post-AMI ruptures as a homogeneous syndrome.

### 4.4. Sex-Related Differences

Women underwent surgery less frequently than men in our cohort despite no statistically significant difference in age and no significant difference in 30-day mortality. This observation is relevant because female sex has repeatedly been associated with both the occurrence and outcome of myocardial rupture. In the large US analysis of STEMI complicated by MCs, women experienced higher in-hospital mortality than men (47.9% vs. 38.2%) [3]. Contemporary US data have also described lower use of CABG and higher adjusted mortality among women admitted with AMI complicated by mechanical rupture [17]. These associations are likely multifactorial and may reflect differences in age, body size, coronary anatomy, infarct characteristics, delayed presentation, comorbidity, shock severity and treatment selection. Our sample is too small to establish whether the lower surgical rate represents a true sex disparity, but the magnitude of the observed difference supports systematic reporting of sex-disaggregated treatment pathways and reasons for nonoperative management in future multicenter studies.

### 4.5. Geographical and Socioeconomic Factors

Socioeconomic disadvantage has been associated with worse outcomes after AMI, including higher mortality and lower use of invasive procedures [18,19]. However, this association appears less consistent when socioeconomic status is assessed using area-level measures; a large US study found no significant association between county-level income and post-AMI mortality [20]. Similarly, area-level income was not associated with 30-day mortality in our cohort.

MCs are particularly suitable for regionalized care because individual hospitals and surgeons may encounter very few cases. Contemporary guidelines and scientific statements consequently emphasize rapid multidisciplinary evaluation and treatment in centers with cardiac surgery, structural intervention, advanced imaging and critical care/MCS expertise [10]. Our patients were referred through a geographically dispersed regional network, yet neither distance to the tertiary center nor area-level income was associated with 30-day mortality. Although statistical power is limited, socioeconomic status was measured ecologically rather than individually, and prehospital deaths are necessarily absent; these findings are reassuring. These findings suggest that an organized *hub-and-spoke* system may help address geographical barriers once a patient with an MC is identified and referred; however, our study was not designed to determine whether regionalized care itself reduces such disparities.

## 5. Limitations

Several limitations should be acknowledged. First, this was a retrospective single-center study with 59 patients, limiting statistical power, precision of subgroup estimates, and the complexity of multivariable modeling. Second, treatment was non-randomized and strongly influenced by clinical status; therefore, associations with surgery or MCS are subject to confounding by indication, survivor bias, and immortal-time bias. Third, MCS strategies evolved over the 10-year study period, and device-specific variables require harmonization before comparative estimates can be reported. Fourth, frailty, baseline functional status, neurological status, treatment limitation decisions, and reasons for withholding surgery or MCS were not systematically captured, limiting interpretation of age- and sex-related treatment differences. Fifth, socioeconomic status was measured ecologically using area-level income rather than individual-level data, and prehospital deaths were necessarily excluded. Finally, the exploratory subgroup analyses should be considered hypothesis-generating and require external validation.

## 6. Conclusions

Post-AMI mechanical complications remain exceptionally lethal in contemporary practice, with 59.3% 30-day all-cause mortality in this 10-year regional cohort. Advanced age was strongly associated with adverse early outcomes. Surgery was associated with lower observed mortality, although causal inference is precluded by treatment selection and survivor bias. The markedly lower use of MCS in patients aged ≥75 years and lower surgical use among women identify clinically important treatment selection patterns that should be examined in larger, prospectively characterized multicenter cohorts. The absence of detectable associations with distance or area-level income after referral supports the potential value of regionalized hub-and-spoke pathways for access to specialized care.

## Figures and Tables

**Table 1:** Baseline characteristics according to 30-day all-cause mortality.

*p* Value	30-Day Survivors (n = 24)	30-Day Deaths (n = 35)	Overall (n = 59)	Characteristic
				Demographics and cardiovascular risk factors
0.002 *	66.0 ± 10.2	74.7 ± 9.5	71.2 ± 10.6	Age, years
0.136	7 (29.2)	17 (48.6)	24 (40.7)	Age ≥ 75 years
0.524	13 (54.2)	16 (45.7)	29 (49.2)	Male sex
0.447	12 (50.0)	14 (40.0)	26 (44.1)	Hypertension
0.800	9 (37.5)	12 (34.3)	21 (35.6)	Dyslipidemia
0.498	3 (12.5)	8 (22.9)	11 (18.6)	Diabetes mellitus
1.000	3 (12.5)	4 (11.4)	7 (11.9)	Obesity
0.138	15 (62.5)	15 (42.9)	30 (50.8)	Smoking history
				Relevant comorbidities
0.509	0 (0.0)	2 (5.7)	2 (3.4)	Atrial fibrillation
0.509	0 (0.0)	2 (5.7)	2 (3.4)	Previous stroke/TIA
1.000	2 (8.3)	4 (11.4)	6 (10.2)	Chronic respiratory disease
0.561	2 (8.3)	1 (2.9)	3 (5.1)	Chronic kidney disease
1.000	1 (4.2)	1 (2.9)	2 (3.4)	Previous coronary artery disease
0.223	1 (4.2)	6 (17.1)	7 (11.9)	Active or previous neoplasia
				Index AMI
0.847	9 (37.5)	14 (40.0)	23 (39.0)	Anterior AMI
0.524	11 (45.8)	19 (54.3)	30 (50.8)	Inferior AMI
				Mechanical complication
0.717	7 (29.2)	10 (28.6)	17 (28.8)	Isolated VSR
	7 (29.2)	14 (40.0)	21 (35.6)	LV free-wall rupture
	6 (25.0)	5 (14.3)	11 (18.6)	Papillary muscle/chordal rupture
	4 (16.7)	6 (17.1)	10 (16.9)	VSR + contained free-wall rupture/pseudoaneurysm
				Management
0.506	21 (87.5)	28 (80.0)	49 (83.1)	Coronary angiography
0.136	17 (70.8)	18 (51.4)	35 (59.3)	Temporary mechanical circulatory support
0.010 *	19 (79.2)	16 (45.7)	35 (59.3)	Definitive invasive treatment †
0.069	3 (12.5)	0 (0.0)	3 (5.3)	Urgent heart -transplant listing
				Geographical and socioeconomic variables
0.666	29.2 [10.2–33.4]	28.2 [17.2–32.2]	28.2 [17.0–32.8]	Distance to referral center, km
0.324	15,951 [14,750–17,413]	15,494 [14,311–16,927]	15,592 [14,531–17,031]	Area-level median income, EUR

Data are presented as n (%), mean ± standard deviation, or median [interquartile range], as appropriate. * Indicates statistically significant *p*-values (*p* < 0.05). † Definitive invasive treatment included surgical repair in 34 patients and percutaneous VSR closure in 1 patient. AMI, acute myocardial infarction; IQR, interquartile range; LV, left ventricular; TIA, transient ischemic attack; VSR, ventricular septal rupture.

## Data Availability

The raw data supporting the conclusions of this article will be made available by the authors on request.

## Supplementary Materials

The following supporting information can be downloaded at https://www.mdpi.com/article/10.3390/jcm15207975/s1, Figure S1: Long-term survival according to sex.
