# Supplementary material for: Mechanical Complications After Acute Myocardial Infarction: Contemporary Management, Treatment Selection, and Outcomes in a 10-Year Regional Referral Network
Source: J Clin Med. 2026 Oct 15;15(20):7975. doi: 10.3390/jcm15207975 (PMC13706350; doi:10.3390/jcm15207975)
Supplement: Supplementary file 1 [file jcm-15-07975-s001.zip › jcm-4612220-supplementary.pdf]

## Supplementary Material Figure S1

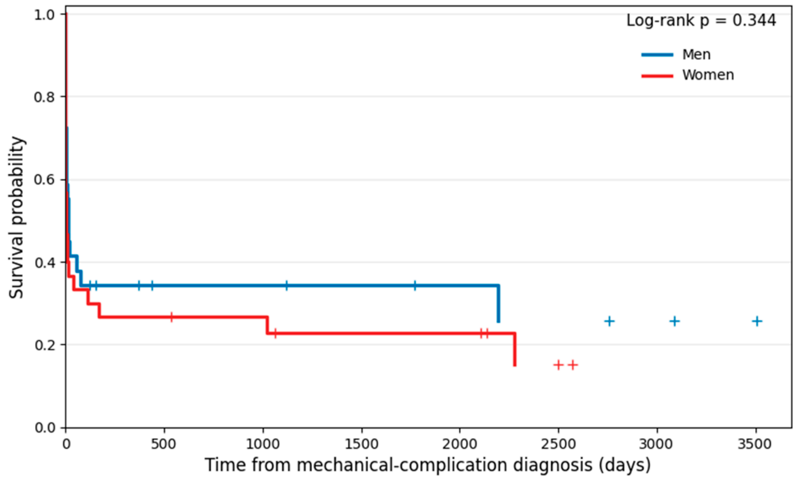


| **No. at risk** | 0 | 1 | 3 | 5 | Years |
|---|---|---|---|---|---|
| Men | 29 | 8 | 6 | 4 | |
| Women | 30 | 8 | 5 | 5 | |

**Supplementary Material Figure S1.** Long-term survival according to sex. Kaplan–Meier estimates of survival from the diagnosis of the mechanical complication, stratified by sex. No significant differences in long-term survival were observed between men and women (log-rank p = 0.344). Censoring is indicated by cross marks, and numbers at risk are shown below the graph.
